# Diabetes knowledge among Malaysian adults: A scoping review and meta-analysis

**DOI:** 10.51866/rv.304

**Published:** 2024-04-27

**Authors:** Pei Kuan Lai, Cheong Lieng Teng, Feisul Idzwan Mustapha

**Affiliations:** 1 Nursing (Hons), MSc in Medical and Health Sciences, PhD in Medical and Health Sciences, Institute for Research, Development and Innovation (IRDI), International Medical University (IMU), No.126, Jalan Jalil Perkasa 19, Bukit Jalil, Kuala Lumpur, Malaysia. Email: laipeikuan@imu.edu.my; 2 School of Medicine, International Medical University (IMU), Clinical Campus Seremban, Jalan Rasah, Bukit Rasah, Negeri Sembilan, Seremban, Malaysia.; 3 Deputy Director (Non-Communicable Diseases), Disease Control Division, Ministry of Health, Malaysia.

**Keywords:** Diabetes mellitus, Health knowledge, Malaysia, Systematic review

## Abstract

**Introduction:**

Optimal self-care promotes glycaemic control and prevents diabetes complications. Its performance is facilitated by an adequate level of diabetes knowledge. This review aimed to evaluate diabetes knowledge among Malaysians by assessing diabetes knowledge scores and their associated factors.

**Methods:**

A comprehensive bibliographic search for Malaysian studies on diabetes knowledge was conducted in PubMed, Scopus and Google Scholar. Relevant literature was systematically selected and described; pertinent data were extracted; and data on diabetes knowledge levels and their associated factors were synthesised. The quality of the identified studies was assessed using a Joanna Briggs Institute critical appraisal tool.

**Results:**

Thirty Malaysian cross-sectional studies that measured diabetes knowledge levels were retrieved. Nineteen of them used a named diabetes knowledge measurement tool, with 14 using the 14-item Michigan Diabetes Knowledge Test. A low knowledge level was prevalent among patients with diabetes mellitus (pooled mean knowledge score=6.92, proportion of patients with a low knowledge level=47.97%). The knowledge score was associated with some sociodemographic variables, health literacy, self-care and glycaemic control.

**Conclusion:**

The association of diabetes knowledge with diabetes outcomes (e.g. self-care and glycaemic control) reflects the potential of the former as a target of intervention. Periodic measurement of diabetes knowledge in healthcare settings and among populations can help in assessing the effectiveness of diabetes educational interventions. Concerted efforts to improve diabetes knowledge among Malaysians have the potential to fill knowledge–practice gaps.

## Introduction

Diabetes mellitus is a major chronic disease that yields increasing burden in low-to-middle-income countries.^1^ In the National Health and Morbidity Survey in 2019, 3.9 million (18.3%) Malaysians aged 18 years and above were found to have diabetes mellitus,^2^ a considerable increase from 13.4% reported in the national survey in 2015.^3^ The National Diabetes Registry (NDR)^4^ recorded 1,614,363 patients with diabetes mellitus from 2013 to 2019, of whom 99.3% were diagnosed with type 2 diabetes mellitus (T2DM). In 2019, the NDR audited 181,634 patients with T2DM from 830 public primary care clinics. A considerable prevalence of comorbidities was reported for these patients: 80.4% for hypertension, 74.3% for dyslipidaemia, 14.6% for nephropathy, 10.6% for retinopathy and 5.9% for ischaemic heart disease. Further, glycaemic targets (>6.5%) were not achieved in 67.6% of these patients.^4^

Diabetes mellitus requires appropriate and persistent self-management, including healthy eating, physical activity, blood glucose monitoring, medication adherence, good problem-solving skills and healthy coping skills.^5^ Better selfcare practice is likely to lead to improved glycaemic control among affected patients, as shown in the systematic review by Chrvala et al.^6^ It has been found that 95% of diabetes care can be achieved by patients with diabetes mellitus themselves.^7^ Although appropriate usage of antidiabetic medications is vital, effective diabetes management also depends on patients’ knowledge about the disease and practice of self-care activities.^8^ Research has shown that more knowledge is associated with a predisposition to assume self-care, leading to reduced stress associated with the disease, higher receptivity to treatment, better trust towards medical professionals, higher selfesteem and self-efficacy and a more positive selfperception of one’s health. ^9,10^

Diabetes knowledge can be measured using various measurement tools. Recognition of baseline diabetes knowledge is important in educating patients with T2DM to help them manage their disease effectively. In other words, assessment of the knowledge level contributes to the design of personalised educational interventions tailored to the individual needs of patients with T2DM. Hence, determination of patients’ knowledge levels and identification of specific knowledge-practice gaps should be first conducted for the implementation of effective and individualised diabetes education.^11^

In a systematic review performed among patients with T2DM in Southeast Asian countries, data on diabetes knowledge were available from only three out of eleven countries.^12^ Out of the seven studies included in this study, only four studies were conducted in Malaysia. However, there were some methodological concerns in this review, as synthesis was conducted although the respondents were variable (patients versus non-patients) and different diabetes knowledge scales were used.^12^

To date, no national study has yet evaluated health knowledge among patients with diabetes mellitus and the general population. There are a substantial number of Malaysian publications on diabetes knowledge; these publications show that the prevalence of low knowledge levels could range from 33.6%^a11^ to 73.5%^a3^ even though the same knowledge questionnaire is used. Thus, a comprehensive review of Malaysian scientific literature is warranted. This review is anticipated to provide data on the types of knowledge assessment tools used and the factors affecting the knowledge levels. The findings could help in the identification of new targets for interventions for better management of patients with and without T2DM in Malaysia. An understanding of the level of diabetes knowledge is also helpful for health educators to plan for future diabetes programmes.

## Methods

An extensive literature search using a combination of the search terms ‘diabetes mellitus’, ‘knowledge’ and ‘Malaysia’ was conducted in electronic bibliographic databases including PubMed and Scopus supplemented by Google Scholar.

The criteria for inclusion of studies to this analysis were as follows: (1) study setting: communities, primary care clinics or hospitals in Malaysia; (2) study participants: adults (≥18 years of age) with diabetes mellitus as well as healthy adults; (3) measurement: general health knowledge about diabetes mellitus; and (4) study design: cross-sectional.

Only original articles were included in the list for analysis. Books, monographs, reports, case reports, conference abstracts, editorials, letters, comments, reviews (narrative or systematic), study protocols and theses or dissertations were excluded from this scoping review. There was no limit set for the year of publication.

The literature search was performed from inception to 17 January 2022. The retrieved references were managed using the EndNote X20 citation manager (London: Clarivate Analytics; 2020). The keywords of all citations were coded for the study designs, study settings (ambulatory care or tertiary care) and diabetes knowledge and its associated factors.

After studies to be included in the analysis were finalised, the full texts of all eligible studies were retrieved. A pair of investigators independently screened the titles and abstracts of eligible studies and reviewed the full texts for methodological validity. When multiple publications of the same study were retrieved, only the most recent relevant data were extracted from these publications. This scoping review was prepared following the PRISMA-ScR checklist.^13^ The quality of the identified studies was assessed using the Joanna Briggs Institute (JBI) Checklist for Prevalence Studies, since it is specifically designed for prevalence studies and has high methodologic rigour.^14,15^ As there is no generally acceptable threshold for an ‘acceptable’ JBI score, studies with a score of 5 or less (suggesting high likelihood of a poor study quality) were excluded.

Subsequently, two investigators extracted and entered the following data independently into a data extraction form: (1) author and year of publication, (2) sample size, (3) participant characteristics, (4) settings, (5) diabetes knowledge measurement tools and (6) key findings.

A meta-analysis of the proportion of the diabetes knowledge scores was performed using the MedCalc statistical software (MedCalc Statistical Software version 20.006. Ostend, Belgium: MedCalc Software Ltd; 2021). A fixed-effect model was selected when the study heterogeneity (I^2^) was less than 50%; otherwise, a random-effect model was used. Further, the MDKT mean scores were synthesised using a cloud-based application provided by rBiostatistics (Dimitri Raptis et al 2024, https://www.rbiostatistics.com/).

The study protocol for this scoping review was registered with the International Platform of Registered Systematic Review and Meta-Analysis Protocols (INPLASY)^16^ under INPLASY registration number INPLASY202190044.

## Results

### Search results

**Figure 1:**
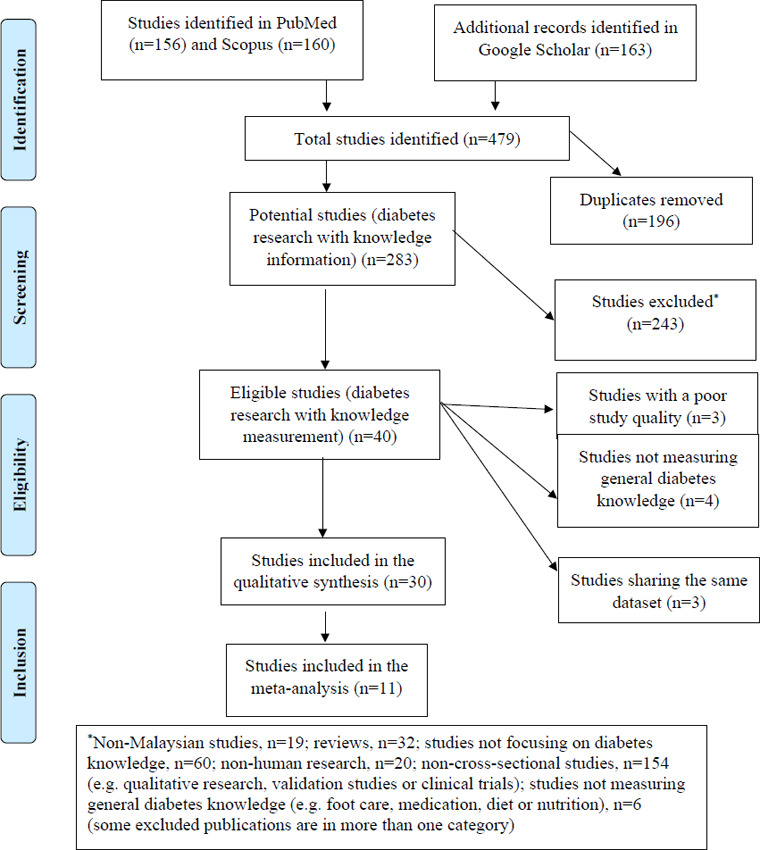
PRISMA-ScR flow diagram.

The online database search yielded 479 results from PubMed (n=156), Scopus (n=160) and Google Scholar (n=163). After 196 duplicates were removed from the list, 283 potential studies were further filtered by reviewing their titles and abstracts. An initial list of 40 publications was considered for analysis after excluding irrelevant studies such as studies not conducted in Malaysia, randomised controlled trials, letters, studies not related to diabetes knowledge, qualitative studies, conference abstracts, study protocols, reviews, non-journal references and interventional studies on the effectiveness of an intervention or a programme (Figure 1). After the full texts were examined, eight publications were further excluded owing to a low JBI score.

### Studies included

Thirty studies published from 2006 to 2021 were included, all of which were cross-sectional studies. Kindly see appendix for the full list of articles.

### Study participants

The majority (n=24, 80%) of the studies involved patients with T2DM, while seven studies (23.3%) involved healthy individuals. Two studies recruited both patients with T2DM and healthy individuals,^a12,a30^ while one study involved both patients with types 1 and 2 diabetes mellitus.^a28^

### Study settings

Twenty-one studies (70%) were conducted in ambulatory care facilities (i.e. public primary care clinics, hospital outpatient clinics or pharmacies), while 10 (33.3%) were conducted in community settings. One study was conducted in hospital and community settings.^a30^ Fifteen studies were performed in primary care settings ^a2-3,a6,a8,a12-14,a17-18,a20-22,a25,a27,a29^ and four of them were also conducted in hospital settings.^a13,a17,a25,a30^

### Study objectives

The objectives of this scoping review included the following: (1) validation of diabetes knowledge measurement tools, (2) evaluation of diabetes knowledge measurement tools, (3) assessment of the prevalence of low knowledge levels among patients with diabetes mellitus, (4) comparison of diabetes knowledge between individuals with and without diabetes mellitus and (5) identification of the factors associated with diabetes knowledge.

***Validity of diabetes knowledge measurement tools***

Two diabetes knowledge scales had been validated in Malaysia and shown to have adequate psychometric properties: Michigan Diabetes Knowledge Test (MDKT, Cronbach’s a=0.702, test–retest reliability=0.894)17 and Orang Asli-Diabetes Knowledge Questionnaire (OA-DKQ, Cronbach’s a=0.806).^a5^

***Diabetes knowledge measurement tools used***

Nineteen studies used a named diabetes knowledge scale (Table 1), while the rest either used a self-developed questionnaire or adapted a tool from various sources. The names of the diabetes knowledge scales used were as follows: (1) MDKT in 15 studies, with all studies using the 14-item version (MDKT-14) with the exception of the study by Abbasi et al.^a1^ where the MDKT-18 was utilised; the total possible MDKT-14 score ranges from 0 to 14 and is interpreted as follows: low (<7 points), moderate (7–10 points) and good (≥11 points); (2) OA-DKQ in two studies, consisting of 12 items and 89 sub-items on general diabetes knowledge; (3) Diabetes Knowledge Scale in one study; and (4) Diabetes Knowledge Questionnaire in one study, consisting of 24 items on general diabetes knowledge.

**Table 1:** Studies using a self-developed or modified diabetes knowledge scale.

No.	Study	Participants	Setting	JBI score	Scale	Diabetes knowledge assessment
1	Abbasi et al. (2018) ^a1^	386 adults with T2DM; mean (SD) age: 54.2 (7.8) years	Pharmacy	7	MDKT-18	Mean (SD) score: 10.5 (4.0) (possible score: 0–18) Approximately 36.8% of participants scored below 60% (cut-off point for a poor knowledge level).
2	Abdullah et al. (2020) ^a2^	427 adults with T2DM; mean (SD) age: 58.1 (10.6) years	Ambulatory care facility	8	MDKT-14	Mean (SD) score: 7.70 (2.31) Knowledge category: NA
3	Abdullah et al. (2019) ^a3^	200 adults with T2DM; mean (SD) age: 60.7 (9.4) years	Ambulatory care facility	8	MDKT-14	Mean (SD) score: 6.1 (2.1) Low knowledge level, 73.5%; moderate knowledge level, 24%; good knowledge level, 2.5%
4	Ahmad et al. (2010) ^a5^	17 healthy indigenous adults	Community	6	OA-DKQ	Validation data only
5	Ahmad et al. (2013) ^a4^	385 healthy indigenous adults; mean (SD) age: 36.4 (14.0) years	Community	7	OA-DKQ	The diabetes knowledge level was poor among the indigenous people in Peninsular Malaysia. Of the 57 sub-items across the eight knowledge questions, only four sub-items (7%) were identified correctly by >50% of the entire cohort.
6	Ahmad et al. (2018) ^a6^	97 adults with T2DM and tuberculosis; mean (SD) age: 56.6 (12.4) years	Ambulatory care facility	7	MDKT-14	Mean (SD) score: 6.89 (1.22)
7	Al-Qazaz et al. (2012) ^a7^	505 adults with T2DM; mean (SD) age: 58.2 (9.2) years	Ambulatory care facility	9	MDKT-14	Mean (SD) score: 7.44 (3.08) Low knowledge level, 41.8%; moderate knowledge level, 46.7%; good knowledge level, 11.5%
8	Azreena et al. (2016) ^a8^	288 adults with T2DM; mean (SD) age: 53.4 (9.9) years	Ambulatory care facility	8	MDKT-14	Mean (SD) score: 7.77 (2.18) Knowledge category: NA
9	Chieng et al. (2015) ^a9^	144 adults with T2DM; mean (SD) age: NA	Hospital and ambulatory care facility	8	MDKT-14	Mean (SD) score: NA Knowledge category: NA
10	Chua et al. (2021) ^a11^	262 adults with T2DM; median (IQR) age: 59 (13.5) years	Ambulatory care facility	9	MDKT-14	Median (IQR) score: 8 (5) Low knowledge level, 33.6%; moderate-to-good knowledge level, 66.4%
11	Gillani et al. (2019) ^a13^	430 adults with T2DM; mean (SD) age: NA	Hospital and ambulatory care facility	9	MDKT-14	Mean (SD) score: 8.22 (1.97) among insulin users and 6.13 (2.29) among OAM users Low knowledge level, 54.4%; moderate-to-good knowledge level, 45.6%
12	Hamidah et al. (2012) ^a15^	109 adults with T2DM; mean (SD) age: NA	Ambulatory care facility	7	MDKT-14	Mean (SD) score: NA Poor knowledge level, 81.7% (score <11); good knowledge level, 17.3%
13	Hasbullah et al. (2021) ^a16^	288 healthy university students; mean (SD) age: 21.7 (1.5) years	University	7	MDKT-14	Mean (SD) score: 7.73 (2.07) among those with a family history of diabetes mellitus and 7.06 (2.26) among those with no family history of diabetes mellitus Low knowledge level, 47.2%; moderate knowledge level, 46.2%; good knowledge level, 6.6%
14	Ishak et al. (2017) ^a17^	143 adults with T2DM; mean (SD) age: 67.9 (5.4) years	Hospital	9	MDKT-14	Mean (SD) score: NA Low knowledge level, 42%; moderate knowledge level, 45.5%; good knowledge level, 12.6%
15	Kueh et al. (2017) ^a19^	266 adults with T2DM; mean (SD) age: 57.0 (8.51) years	Ambulatory care facility	7	DKS	Mean (SD) score: 52.5 (17.01) (possible score: 0–100)
16	Lee et al. (2019) ^a20^	196 adults with T2DM; mean (SD) age: NA	Ambulatory care facility	9	MDKT-14	Mean (SD) score: 6.8 (2.6) Low knowledge level, 42.9%; moderate knowledge level, 53.6%; good knowledge level, 3.6%
17	Qamar et al. (2017) ^a23^	350 healthy adults; mean (SD) age: NA	Community	6	DKQ	Mean (SD) score: 11.11 (6.09) Low knowledge level, 32.0%; moderate knowledge level, 41.7%; good knowledge level, 26.3%
18	Tam et al. (2014) ^a27^	770 healthy adults; mean age (SD): 30.7 (13.0) years	Community	9	MDKT-14	Mean (SD) score: 6.20 (2.15) Knowledge category: NA
19	Yap et al. (2014) ^a29^	187 adults with T2DM; mean (SD) age: NA	Ambulatory care facility	6	MDKT-14	Mean (SD) score: NA Knowledge category: NA

T2DM, Type 2 diabetes mellitus; NA, not available; IQR, Inter-quartile range; SD, standard deviation; DKS, Diabetes Knowledge Scale; DKQ, Diabetes Knowledge Questionnaire; MDKT, Michigan Diabetes Knowledge Test; OA-DKQ, Orang Asli-Diabetes Knowledge Questionnaire; OAM, oral antidiabetic medication.

Eleven studies used adapted/modified or self-developed questionnaires to measure diabetes knowledge (Table 2). There were marked variations in the contents covered, with the number of items varying from 11 to 68 scored using different methods. Only three studies reported psychometric data prior to data collection.^23,28,44^

**Table 2:** Studies using a self-developed or modified diabetes knowledge scale.

No.	Study	Participants	Setting	JBI score	Diabetes knowledge assessment
1	Chinnappan (2017) ^a10^	400 healthy individuals aged >12 years	Community	7	11 items covering general awareness, symptom, complication, prevention and control; mean knowledge score not reported; no psychometric data shown
2	Ding et al. (2006) ^a12^	149 adults: 83 patients with T2DM (mean age: 53.3 years) and 66 patients without T2DM (mean age: 34.5 years)	Ambulatory care facility	7	41 items covering general knowledge, risk factors, symptoms and complications, treatment and management and monitoring; mean (SD) knowledge score: 81.8% (10.9) among those with diabetes mellitus and 64.0% (20.9) among those without diabetes mellitus; no psychometric data reported
3	Gunggu et al. (2016) ^a14^	400 adults with T2DM; mean (SD) age: 58.77 (11.46) years	Ambulatory care facility	6	11 items (details not provided); mean (SD) knowledge score: 8.31 (1.82); no psychometric data shown
4	Kang et al. (2018) ^a18^	546 adults with T2DM; mean (SD) age: NA	Ambulatory care facility	7	13 items covering complications (six items) and risk factors (seven items); median (IQR) score for diabetes complications: 5 (1); median (IQR) score for risk factors: 6 (2); questionnaire validation revealing a Cronbachs a of 0.566–0.917
5	Ng et al. (2012) ^a21^	75 adults with T2DM; mean (SD) age: 59.2 (11.6) years	Ambulatory care facility	6	14 items, content unclear; mean (SD) knowledge score: 11.85 (2.45); no psychometric data reported
6	Noor Azimah et al. (2010) ^a22^	110 adults with T2DM; mean (SD) age: 58.3 (10.4) years	Ambulatory care facility	6	68 items covering nature, risk factors, complications, self-care and nutrition; mean (SD) knowledge score: 71.2% (9.34); no psychometric data shown
7	Remali et al. (2019) ^a24^	68 healthy adults; median (IQR) age: 26.0 (19.8) years	Community	6	25 items covering general knowledge; poor knowledge level (<13 points), 1.5%; moderate knowledge level (14–18 points), 54.5%; good knowledge level (>18 points), 44.1%; no psychometric data shown
8	Shahar et al. (2019) ^a25^	276 adults with T2DM; median (IQR) age: 56 (48–62) years	Ambulatory care facility and hospital	6	18 items, content unclear; median (IQR) knowledge score: 0.70 (0.50–0.88); no psychometric data reported
9	Sulaiman et al. (2020) ^a26^	336 healthy adults; mean (SD) age: 22.00 (3.96) years	University	6	35 items covering general knowledge, risk factors, symptoms, prevention and complications; median knowledge score (≥22 points): 52.7%; questionnaire validation revealing a Cronbach’s α of 0.62
10	Tan et al (2008) ^a28^	126 adults with T1DM and T2DM; mean (SD) age: 54.7 (11.06) years	Hospital and ambulatory care facility	7	20 items covering general knowledge, symptoms, diet and complications; mean (SD) knowledge score: 8.6 (3.6); 53% of participants scoring below 50%; questionnaire validation revealing a Cronbach’s α of 0.71
11	Yun et al. (2007) ^a30^	120 adults with T2DM; mean (SD) age: 55.57 (8.73) years 120 healthy adults; mean (SD) age: 53.67 (7.90) years	Ambulatory care facility and community	6	30 items covering general knowledge (six items), risk factors (four items), symptoms and complications (nine items), treatment and management (nine items) and monitoring (two items); mean (SD) knowledge score: 24.4 (3.83) among patients with diabetes mellitus and 20.2 (5.97) among healthy individuals; no psychometric data shown

***Diabetes knowledge level among patients with diabetes mellitus***

Of the 15 studies that used the MDKT, all except one^a1^ used the MDKT-14. Of these 14 studies using the MDKT-14, 11 reported the diabetes knowledge level using means or proportions. Two studies did not report the above-summarised data,^a9,a29^ and one study used a different threshold for the low knowledge level (score <11).^a15^ The 14 studies had JBI scores ranging from 6 to 9.

**Figure 2:**
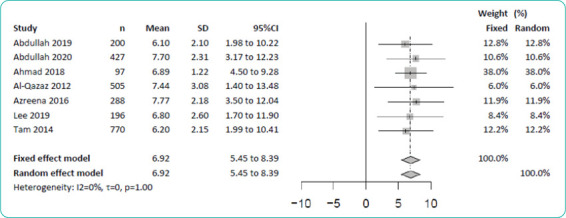
Meta-analysis of the mean Michigan Diabetes Knowledge Test (MDKT) scores.

In view of the similarity of the measurement tool used (MDKT-14), the extracted data were synthesised. As shown in Figure 2, seven studies involving 2483 participants reported mean MDKT-14 scores ranging from 6.10 to 7.77 with SDs ranging from 1.22 to 3.08. A metaanalysis of these data (fixed-effect model) revealed a pooled mean score of 6.92 (95% confidence interval [CI] = 5.45–8.39). When the study by Tam et al.^a27^ (healthy adults) was excluded to limit the meta-analysis to only patients with diabetes mellitus, the pooled mean knowledge score was 7.02 (95% CI=5.45-8.60).

**Figure 3:**
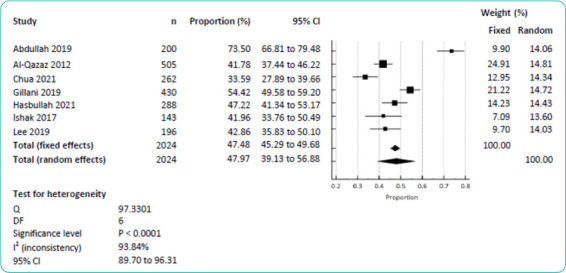
Meta-analysis of the proportion of a low diabetes knowledge level.

As shown in Figure 3, seven studies involving 2024 participants reported the numbers or proportions of participants having a low knowledge level. A meta-analysis of these data (randomeffect model) revealed a pooled proportion of a low knowledge level of 47.97% (95% CI=39.13–56.88). When the study by Hasbullah et al.a16 (healthy adults) was excluded to limit the metaanalysis to only patients with diabetes mellitus, the pooled proportion was 48.10% (95% CI=37.61-58.68).

***Diabetes knowledge between individuals with and without diabetes mellitus***

Two studies assessed diabetes knowledge among individuals without diabetes mellitus using the MDKT-14: Tam et al.^a27^ reported a mean (SD) knowledge score of 6.20 (2.15), while Hasbullah et al.a16 reported a proportion of a low knowledge level of 47.22% (95% CI=41.34–53.17) (Figures 2 and 3). Conversely, two studies compared diabetes knowledge between individuals with and without diabetes mellitus using the same scale simultaneously.^a12,a30^ Ding et al.^a12^ found that the overall knowledge score was significantly higher among patients with diabetes mellitus (81.8, SD=10.9) than among patients without diabetes mellitus (64.0, SD=20.9). Yun et al.^a30^ showed that the mean total knowledge score was significantly higher among patients with diabetes mellitus (24.4, SD=3.83) than among healthy individuals (20.2, SD=5.97).

***Factors associated with the diabetes knowledge level***

There was considerable heterogeneity among the included studies, particularly in relation to the diabetes knowledge measurement tools used. In many studies, diabetes knowledge was not the main outcome measure.

In terms of sociodemographic data, Al-Qazaz et al.^a7^ reported that a lower educational level and an older age, but not sex and ethnicity, were associated with a lower knowledge score (P<0.05) among patients with diabetes mellitus. Chua et al.^a11^ showed that an Indian ethnicity, a lower household income and a lower educational level were significantly associated with a lower knowledge score (P=0.003, P=0.027 and P<0.001, respectively). Lee et al.^a20^ demonstrated that an Indian ethnicity and a lower educational level were significantly associated with a lower knowledge score (P=0.005 and P<0.001, respectively).

In terms of health literacy, Abdullah et al.^a2^ used the HLS-EU-Q47 to measure this variable. They found that patients with adequate health literacy had a higher MDKT-14 score (P=0.019).

In terms of glycaemic control, Abdullah et al.^a3^ failed to observe an association between the HbA1c level and MDKT-14 score. In contrast, Al-Qazaz et al.^a7^ found a significant linear correlation between the HbA1c level and total knowledge score (r=-0.39, P<0.001).

In terms of self-care, Ishak et al.^a17^ used the Malay Elderly Diabetes Self-Care Questionnaire. The authors reported that better diabetes knowledge was associated with self-care (P<0.001). Yap et al.^a29^ found a linear correlation between self-care based on the Summary of Diabetes Self-Care Activities questionnaire score and diabetes knowledge (r=0.28, P=0.001).

## Discussion

This scoping review of Malaysian literature evaluated 30 cross-sectional studies that measured diabetes knowledge over a 16-year period (from 2006 to 2021). Various measurement tools for diabetes knowledge were used across these studies. The MDKT, which was originally developed by Fitzgerald et al,^18^ was most frequently used by Malaysian diabetes researchers in view of acceptable psychometric data in the local setting.^17^ The MDKT was also noted to be commonly used in two other reviews: a systematic review of diabetes knowledge in Saudi Arabia^19^ and a systematic review of dietary knowledge measurement instruments for diabetes.^20^

Given the wide variation in the contents of the diabetes knowledge measurement tools used among the studies reviewed in this study, it was inappropriate to compare the mean score or proportion between these studies. By limiting the analysis only to the studies that used the MDKT-14, the metaanalysis yielded a more credible pooled mean knowledge score of 6.92 (95% CI=5.45–8.39) and a proportion of a low knowledge level of 47.97% (95% CI=39.13-56.88). This synthesis showed that the mean knowledge score of Malaysian patients with diabetes mellitus is relatively low, with almost half of them having a low diabetes knowledge level (score <7).

The findings of this scoping review are consistent with those of systematic reviews conducted in other countries.^21-23^ Although these systematic reviews used somewhat different methodologies, they also found that patients with diabetes mellitus generally had relatively low knowledge scores. Among the Malaysian studies that utilised the MDKT-14, there were associations found between a low knowledge level and certain sociodemographic variables such as Indian ethnicity, older age, lower educational level and lower household income. Notably, these variables are closely related to poverty and social disadvantages and are not amenable to change by healthcare providers.

In the present study, diabetes knowledge was found to be positively associated with health literacy, various self-care activities and glycaemic control (i.e. lower HbA1c levels). The positive relationship between diabetes knowledge and practice-level outcome is reassuring, as this supports the notion that better diabetes knowledge, probably acting in concert with health literacy and self-efficacy, could lead to better diabetes outcomes.^7,24^ Diabetes knowledge is considered easier to measure among patients with diabetes mellitus than health literacy or self-efficacy, wherein reliable responses require relatively high general literacy levels.

Since diabetes knowledge may be a surrogate marker of health literacy or self-efficacy as well as an immediate outcome of diabetes education, measuring it periodically during long-term care of patients with diabetes mellitus should be considered. Diabetes knowledge among the wider population should be assessed to identify specific areas of knowledge gaps that deserve closer attention for future educational activities.^25^ There is currently no attempt to measure diabetes knowledge at the population level in Malaysia within the nationwide Health and Morbidity Surveys. The Malaysian Diabetes Index in 2021,^26^ a nationwide survey of 2539 adults, found that Malaysians had an unsatisfactory level of understanding about diabetes mellitus and were often misinformed about the disease (e.g. one in three Malaysians believed that cutting down sugar is sufficient to prevent diabetes mellitus). This finding shows the urgency of assessing diabetes knowledge, which can provide useful information to inform the effectiveness of diabetes education on general adults as well as patients with diabetes mellitus.

Notably, having good diabetes knowledge may not automatically translate into appropriate actions at the patient level.^27^ This common occurrence of knowledge-action gaps points to the need to measure knowledge as well as specific diabetes mellitus-specific actions such as appropriate self-care practices and work towards reducing barriers to these practices.

Some limitations of this review must be considered. In view of the heterogeneity in the methodologies and objectives across the studies, only a small subset of the studies that used the MDKT reported extractable data for the meta-analysis, reducing the precision of the pooled data (i.e. wider CIs). Further, this review included only cross-sectional studies that measured diabetes knowledge in Malaysia. The findings on the associated factors of diabetes knowledge were derived from these cross-sectional studies and thus lack the predictive value that can be obtained only from long-term cohort studies or randomised controlled trials.

## Conclusion

This study reviewed fairly extensive research on diabetes knowledge in Malaysia and highlighted a suboptimal knowledge level among adults with and without diabetes mellitus. The results of this review can assist Malaysian diabetes researchers in planning further research regarding diabetes knowledge.

## Review protocol registration

The study protocol for this scoping review was registered with the International Platform of Registered Systematic Review and Meta-Analysis Protocols (INPLASY) under INPLASY registration number INPLASY202190044.
